# New Perspectives in Nutritional Management for Neonatal Health

**DOI:** 10.3390/nu18081251

**Published:** 2026-04-16

**Authors:** Silvia Salvatore, Mariella Baldassarre, Maria Lorella Giannì, Massimo Agosti

**Affiliations:** 1 Department of Medicine and Technological Innovation, Pediatrics, University of Insubria, Hospital “F. Del Ponte”, 21100 Varese, Italy; 2 Department of Interdisciplinary Medicine, Neonatology and NICU, Aldo Moro University, 70121 Bari, Italy; mariaelisabetta.baldassarre@uniba.it; 3 Fondazione IRCCS Ca’ Granda Ospedale Maggiore Policlinico, NICU, Via Commenda 12, 20122 Milan, Italy; maria.gianni@unimi.it; 4 Department of Clinical Sciences and Community Health, University of Milan, 20122 Milan, Italy; 5 Department of Medicine and Surgery, Pediatrics, University of Insubria, Hospital “F. Del Ponte”, 21100 Varese, Italy

## 1. Introduction

The neonatal period and early childhood events are increasingly emerging as key determinants of human health. The first 1000 days of life have a crucial influence on brain development, behavior, mood, and cardiovascular, metabolic, allergic, autoimmune, inflammatory, and gastrointestinal disorders [1,2]. The interaction between genes, nutrition, environment, microbiota, exposure to stress, various substances and medications, infections, and traumatic events can influence individual disease susceptibility [3]. Nutritional management is of fundamental importance for neonatal health, particularly for preterm and low-birth-weight infants, who are at significantly higher risk of morbidity and mortality than full-term infants. Adequate early nutritional support is essential to prevent adverse health outcomes, promote normal growth, improve resistance to infections, and optimize neurodevelopmental outcomes [4]. The consequences of suboptimal nutrition extend beyond the neonatal period. Early growth restriction and inadequate nutrition are established risk factors for neurodevelopmental disorders and cardiometabolic diseases in adulthood [5,6]. Conversely, optimal nutrition in early childhood offers the opportunity to improve long-term outcomes for children and the overall health of future generations. Infant nutrition, particularly for those born prematurely, has progressed significantly in recent years [7]. Numerous interventions and preventive strategies have been explored, and current research is focusing on personalized and precision medicine starting in early childhood.

The *Nutrients* Special Issue “Nutrition Management in Neonatal Health” (https://www.mdpi.com/journal/nutrients/special_issues/1LU4P28816) (8 March 2026) collected 13 papers (including original studies, reviews, and meta-analyses) highlighting recent evidence and new developments in the role of nutrition during this vulnerable and crucial period of life.

## 2. Overview of the Published Papers, Emerging Findings, and Research Gaps

Van Neerven [van Neerven, contribution 1] provides a comprehensive review of the effects of malnutrition (including nutritional deficiencies and obesity) on the immune system. The article explores breastfeeding and the bioactive components of infant formula, as well as the immune mechanisms of macronutrients, micronutrients, and other dietary components particularly important for the development and function of the immune system in early childhood. Micronutrient deficiencies (e.g., vitamin D, vitamin A, zinc, and iron) are widespread globally, even in the absence of visible signs. Food influences immunity in multiple ways: by shaping the gut microbiota, interacting with intestinal barriers, and directly influencing immune cells [2,8,9]. In this context, nutrition in early childhood is crucial [10]. Maternal nutrition influences the infant’s immune system during pregnancy and breastfeeding, providing essential nutrients, antibodies, and bioactive compounds that protect against infections and support immune development. When breast milk is not available, infant formula should provide all necessary macronutrients and micronutrients, as well as additional components beneficial to the immune system. The gut microbiota plays a key role in immune development, especially during infancy [2]. Breastfeeding promotes the presence of beneficial bacteria that produce short-chain fatty acids, which support immune balance and reduce inflammation. Other dietary components, such as polyunsaturated fatty acids, plant compounds (polyphenols and flavonoids), and fiber, also contribute to a healthy immune system. A balanced diet, a healthy gut microbiota, and gradual introduction of foods help strengthen the immune system; reduce the risk of infections, inflammation, and allergies; and promote long-term health [11,12].

The review by Chen et al. [Chen, contribution 2] emphasizes that nutrition plays a pivotal role in the care of preterm infants, particularly those at risk for complications such as bronchopulmonary dysplasia, necrotizing enterocolitis, metabolic bone disease, patent ductus arteriosus, and retinopathy of prematurity. Adequate energy intake is essential, starting with modest caloric intake after birth and gradually increasing to higher levels to support growth, especially in infants with increased metabolic needs such as those with bronchopulmonary dysplasia. At the same time, fluid management must be carefully balanced, as excessive fluid intake can worsen patent ductus arteriosus and respiratory outcomes, while overly restrictive strategies can compromise growth and metabolic stability. Early initiation of enteral nutrition is strongly recommended, as it reduces dependence on parenteral nutrition, which is associated with infections and other complications. Breast milk is universally recognized as the optimal nutritional source due to its protective bioactive components, while donor milk is an acceptable alternative when breast milk is unavailable, although it is often necessary to enrich it to meet the increased nutritional needs of preterm infants. An adequate macronutrient intake is crucial: proteins support tissue growth and lung development, carbohydrates provide essential energy but must be controlled to avoid hyperglycemia, and lipids offer a dense energy source suitable for infants with fluid restriction, while also contributing to anti-inflammatory processes. Micronutrients also play an important role, particularly vitamin A, in lung development, although evidence supporting high-dose supplementation remains inconclusive. Other nutrients, such as vitamin D, zinc, and selenium, contribute to immune function and tissue repair. In the prevention and management of necrotizing enterocolitis, early and carefully increased enteral feeding, the use of breast milk, and selected probiotics are beneficial. Parenteral nutrition remains necessary during the acute phase of the disease but should be minimized whenever possible. Gradual reintroduction of enteral feeding after recovery is recommended to promote intestinal adaptation. Metabolic bone disease of prematurity is primarily caused by insufficient calcium and phosphorus intake rather than vitamin D deficiency, highlighting the importance of adequate mineral supplementation and its monitoring. For retinopathy of prematurity, early and adequate nutrition promotes growth and insulin-like growth factor 1 levels, which are important for retinal development, while avoiding hyperglycemia is essential. Breast milk and a balanced intake of fatty acids can further reduce risk. Overall, the review emphasizes that early enteral feeding with breast milk, careful nutrient introduction, appropriate supplementation, and individualized management of fluids and metabolic parameters are key strategies for improving outcomes in preterm infants.

The retrospective study “Fetal Distress as a Determinant for Refeeding Syndrome in Preterm Neonates” by Di Chiara et al. [Di Chiara, contribution 3] investigated the clinical determinants and outcomes of Refeeding Syndrome (RS) in 412 preterm neonates (gestational age below 34 weeks or weight below 1500 g). The authors acknowledged the lack of a universally accepted definition for neonatal RS, which may limit the comparability of these findings with other neonatal populations. Interestingly, the authors identified fetal distress as a primary prenatal risk factor for RS and found a higher prevalence of extrauterine growth restriction and retinopathy of prematurity. The study features a robust sample size and provides novel evidence linking prenatal hypoxia (fetal distress) to metabolic instability in the postnatal period. The use of blinded observers and statisticians enhances the reliability of the reported morbidities, although the retrospective nature introduces potential selection bias and incomplete data collection. Furthermore, the higher caloric intake in the RS group suggests that “aggressive” nutritional protocols themselves remain a major confounding factor that requires more nuanced stratification.

Specific nutritional interventions demonstrate measurable benefits. Micronutrient supplementation, including vitamin A, iron, zinc, calcium, phosphorus, and vitamin D, reduces anemia and mortality rates with minimal side effects [4]. Iron deficiency is a common micronutrient deficiency worldwide, especially in premature infants. During the neonatal period and early childhood, an optimal iron status has positive implications for growth, neurological development, and immune function. A better understanding of iron absorption and regulation, the risk of deficiency/toxicity, and the need for and timing of iron supplementation is important for improving well-being at the individual and population levels [13].

The cross-sectional study “Is Iron Supplementation Associated with Infant Mortality in Sub-Saharan Africa and Does Birth Weight Modify These Associations?” by Bekele et al. analyzes a massive dataset of 287,642 neonates and 279,819 post-neonates across 26 sub-Saharan countries [Bekele, contribution 4]. Maternal iron supplementation for ≥90 days was associated with a reduction by 27% of neonatal mortality and by 45% of post-neonatal mortality and improved survival of infants with low birth weight (LBW). A lack of iron supplementation increased the odds of neonatal mortality by 68% and of postnatal mortality by 25%. The study’s primary strength is its unprecedented scale and the use of stratified analysis, highlighting that LBW infants are a uniquely vulnerable subgroup that benefits most from maternal iron. The use of generalized linear mixed models appropriately accounted for the hierarchical clustering of the data. The heavy reliance on subjective maternal recall for medical data and the lack of clinical biomarkers limit the precision of the findings. Additionally, the non-significant result for overall neonatal mortality in the adjusted model suggests that antenatal care utilization may be a major confounding variable that was difficult to isolate. The retrospective study “Maternal Serum Ferritin Levels in Third Trimester and Risk of Small for Gestational Age in Northern Thailand: Implications for Management in Pregnancy” by Thaichana et al. [Thaichana, contribution 5] explored the dose–response relationship between maternal serum ferritin levels in late pregnancy (30–34 weeks) and adverse birth outcomes in 362 neonates. The authors provided compelling evidence that elevated ferritin levels are significantly associated with a 3.31-fold higher risk of infants being Small for Gestational Age (SGA) and having reduced placental weight. The study identifies a clear, dose-dependent relationship between ferritin and SGA, supported by robust statistical methods including restricted cubic spline models. The focus on third-trimester timing is a significant strength, as it captures the period of maximal fetal growth. The primary weaknesses are the use of historical data (1989–1990) and the lack of control for inflammation, which may confound the interpretation of ferritin as a pure marker of iron status. These factors necessitate caution when translating the findings for individualized nutrition management.

Breast milk is the cornerstone of optimal neonatal nutrition. For very preterm infants, human milk is the best but often requires fortification to meet their enhanced nutritional demands and support adequate growth [7,14]. Reyes et al. [Reyes, contribution 6] performed a systematic review and meta-analysis on the impact of human milk-derived fortifiers on necrotizing enterocolitis (NEC) in very low birthweight (VLBW) infants. Analyzing 20 studies (five randomized controlled trials, RCTs, and 15 observational studies) involving 6794 infants, the authors demonstrated that an exclusive human milk diet was associated with significantly lower odds of both medical NEC (Bell Stage 1–2) and surgical NEC compared to diets containing cow’s milk products. The study uses a comprehensive search strategy and a random-effects model that accounts for study variability. Its primary strength is the consistency of effect estimates across both randomized and observational designs, which increases confidence in the real-world effectiveness of an exclusively human milk diet. The review is limited by the small number of available RCTs and the inherent risk of bias in retrospective observational data, such as confounding by indication. Furthermore, the results are primarily from high-income countries, limiting their generalizability globally. Farella et al. [Farella, contribution 7] conducted a narrative review on how the complex interactions among human milk components modulate infants’ bone health. They emphasize that advances in omics technologies have enabled a deeper characterization of human milk composition and a better understanding of the mechanisms through which it influences bone development and health. Current evidence indicates that different components can act on the same pathways and exert both direct and indirect effects on bone health, underscoring their synergistic interactions. Proteins like casein and whey enhance calcium absorption and bone formation, while fatty acids such as docosahexaenoic acid (DHA) help regulate inflammation and bone breakdown. Human milk oligosaccharides (HMO) improve microbiota composition and calcium availability, contributing to bone mineralization and gut health. Hormones like insulin-like growth factor 1 (IGF-1), leptin, and adiponectin influence growth, body composition, and bone density, and microRNAs play a role in regulating fat and bone development. This complex interplay between nutrition and infant health is also investigated by Argüelles-López et al. [Argüelles-López, contribution 8] in a prospective longitudinal study of 27 mothers and their exclusively breastfed infants. The authors examined how breast milk shapes the gut microbiota and how their characteristics and maternal nutritional status are closely interrelated and associated with infants’ weight-gain trajectories. About half of the infants showed normal weight gain in both the first and second semesters of life. Higher breast milk protein intake was linked to normal weight gain early on and was inversely related to maternal BMI. At 5 months of age, the infant gut microbiota was mainly composed of Veillonella, Bifidobacterium, and Escherichia–Shigella, with fat and oligosaccharides associated with specific bacterial groups (Veillonella abundance and Lachnospiraceae, respectively), while at 12 months, Bacteroides became dominant. Infants with normal weight gain tended to have more Bifidobacterium, whereas those with rapid or slow weight gain showed higher levels of other bacteria such as Ruminococcus gnavus and Alistipes. Their findings further underscore the key role of breastfeeding in modulating the risk of obesity later in life through different synergistic mechanisms.

Preterm infants, particularly those born before 28 weeks’ gestation, require exceptionally high intakes of energy, protein, and other essential nutrients to match the rapid fetal growth that would occur in utero [7]. In very preterm infants, parenteral nutrition is commonly initiated within 24 h of birth, with amino acid intakes of up to 3.5 g/kg per day [7]. Kim et al. [Kim, contribution 9] analyzed perinatal and postnatal data from 2490 very-low-birth-weight (VLBW) infants to study the relationship between the duration of parenteral nutrition and the development of specific comorbidities. Extended parenteral nutrition beyond 28 days was associated with an increased risk and severity of periventricular leukomalacia and bronchopulmonary dysplasia. These findings highlight the need to encourage shorter durations of parenteral nutrition to prevent specific comorbidities in this vulnerable population. Building on the widely recognized importance of nutrition in reducing the risk of comorbidities, particularly in the most fragile neonatal populations, Thoene et al. [Thoene, contribution 10] conducted a retrospective study of 61 extremely low-birth-weight (ELBW) infants to examine how the timing (in hours) of enteral feeding relates to clinical outcomes. Their results demonstrate the feasibility of early enteral feeding in ELBW infants and its association with improved respiratory outcomes and growth and shorter hospital stays. Notably, the role of nutrition as an epigenetic modulator of health outcomes is not limited to a single stage of life; maternal nutrition remains crucial throughout the lifespan and is even more complex during pregnancy, when additional layers of biological communication between the mother and fetus emerge. In line with this evidence, González-Ludlow et al. [González-Ludlow, contribution 11] evaluated the association between maternal folate and vitamin B12 status during pregnancy in 90 healthy mothers and neonatal growth outcomes, including birth weight, length, waist circumference, and percentage of fat mass. Based on their findings, the authors emphasize the importance of maintaining an optimal and balanced maternal B-complex vitamin status during pregnancy to prevent potential growth alterations resulting from excessive or insufficient exposure to these micronutrients, which could negatively impact both short- and long-term outcomes. In the multicenter observational study GREEN MOTHER, which involved 393 mothers who responded to an infant feeding questionnaire and a 24 h dietary history, Cabedo-Ferreiro et al. [Cabedo-Ferreiro, contribution 12] found that maternal diet in the postpartum period did not differ significantly based on infant feeding type, with the exception of a slightly higher intake of polyunsaturated fatty acids (PUFAs) in breastfeeding mothers compared to mothers of formula-fed infants. While breastfeeding mothers have higher nutritional needs, exclusively breastfeeding mothers have a higher median energy intake, often consuming approximately 200 kcal more than non-breastfeeding mothers. Despite this, energy intake was insufficient in 57% of all participants. No significant differences in intake of most macronutrients and energy were found between exclusively breastfeeding mothers, mixed-feeding mothers, and formula-feeding mothers. Regardless of feeding type, most mothers did not meet the recommended daily intake (RDI) for micronutrients such as iron, vitamin D, calcium, and magnesium. Conversely, they were found to consume excessive amounts of refined carbohydrates and simple sugars, primarily from sugar-sweetened beverages. Therefore, the GREEN MOTHER study emphasizes the need for personalized nutrition education for mothers during pregnancy and the postpartum period, as many experience nutritional deficiencies and follow unhealthy diets.

The necessity, timing, and duration of eliminating cow’s milk protein from the diets of neonates and infants with rectal bleeding associated with food protein-induced allergic proctocolitis (FPIAP) are still a matter of debate [15,16,17,18]. On the one hand, because the allergy is often transient, when mild rectal bleeding occurs in an otherwise healthy infant, maternal (if breastfed) or infant dietary changes are often postponed or deemed unnecessary, in part because of the potential risk of the children becoming hyperallergic. On the other hand, in addition to the stress experienced by parents due to the presence of blood in their infant’s stool and the early cessation of breastfeeding, persistent mucosal inflammation with erosions and bleeding can lead to anemia, impaired intestinal barrier function, and an increased predisposition to the development of allergies [14,19,20]. Scavella et al. [Scavella, contribution 13] analyzed neonatal characteristics, clinical presentation, and allergic outcomes in two Italian cohorts, including 94 infants, 62% of whom were exclusively breastfed and 19% were born preterm. Late acquisition of tolerance to cow’s milk was associated with preterm birth, breast milk fortification, early antibiotic exposure, growth retardation, and recurrent infections. Logistic regression identified significantly increased risk odds values for family history of atopy (OR 5.4), atopic dermatitis (OR 8.2), rectal bleeding > 18 days before the elimination diet (OR 5.9), and IgE sensitization (OR 6.4) as potential predictors of late acquisition of tolerance to cow’s milk. These findings, if confirmed in a large, prospective, multicenter study, could guide clinicians to promptly consider an elimination diet and prolonged monitoring in infants with FPIAP and the aforementioned associated risk factors, leading to a personalized approach to diet in this population.
